# A Giant Oncocytoma in an Elderly Female Patient: A Case Report

**DOI:** 10.7759/cureus.41612

**Published:** 2023-07-09

**Authors:** Zisis Kratiras, Georgios Kotronopoulos, Aris Kaltsas, Evangelos Fragkiadis, Konstandinos Stravodimos

**Affiliations:** 1 Department of Urology, Attikon University Hospital, Athens, GRC; 2 Department of Urology, Laiko General Hospital, Athens, GRC; 3 Department of Urology, University of Loannina, Loannina, GRC

**Keywords:** nephrectomy, surgical management, benign renal tumor, renal neoplasm, oncocytoma

## Abstract

Renal oncocytomas are rare, benign tumors that can be difficult to distinguish from malignant renal cell carcinomas. This case report presents an 84-year-old woman with a sizeable renal oncocytoma and discusses this rare entity's diagnostic challenges and management.

## Introduction

Renal oncocytomas are benign epithelial tumors arising from the intercalated cells of the renal collecting ducts. These tumors account for 3-7% of all solid renal tumors and usually have an indolent clinical course. Although most renal oncocytomas are small, unilateral lesions, some cases of large oncocytomas have been reported. Differentiating oncocytomas and renal cell malignancies is difficult because of overlapping clinical, radiologic, and histopathologic features. We present a case of giant renal oncocytoma in an 84-year-old woman and discuss the diagnostic challenges and management.

## Case presentation

An 84-year-old woman with a history of smoking, hypertension, and mild chronic obstructive pulmonary disease was referred to our department for painless frank hematuria. Physical examination revealed mild abdominal tenderness and a palpable mass suggestive of a classic Virchow sign (pain, hematuria, and palpable mass). Diagnostic workup included laboratory tests, urine cytology, flexible cystoscopy, and ultrasonography of the kidneys and urinary bladder (KUB). Laboratory tests were within normal range, cystoscopy was unremarkable, and urine cytology was negative for malignancy.

**Figure 1:**
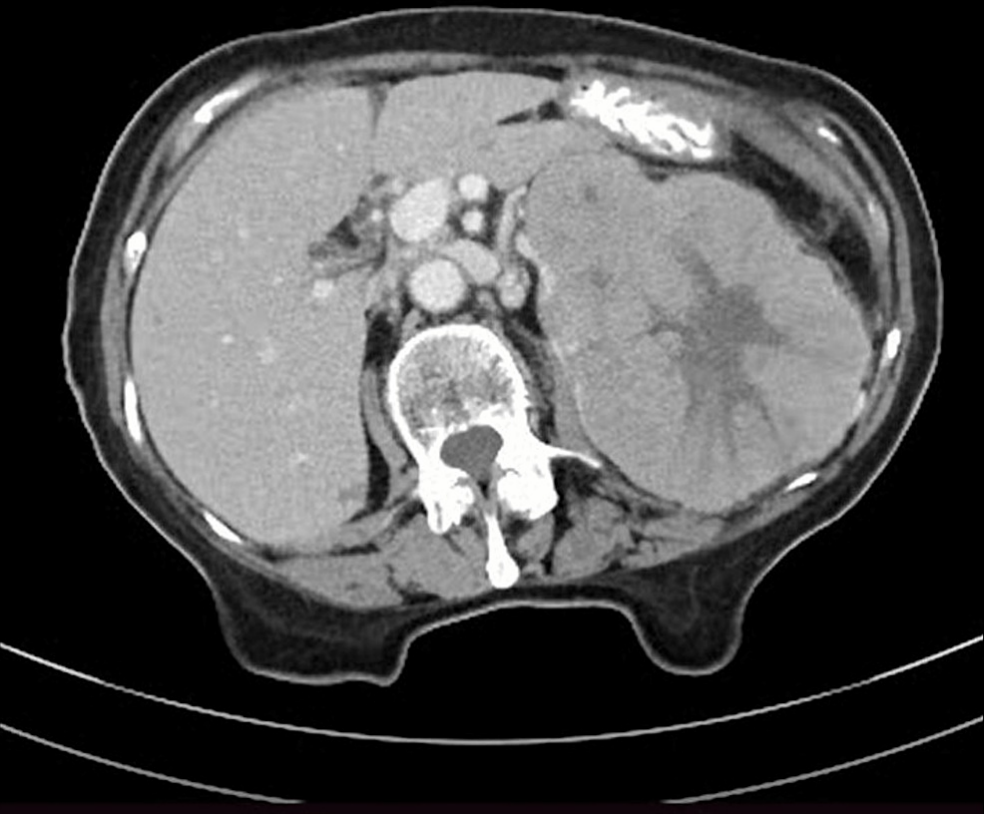
CECT of the abdomen demonstrating a large left renal mass, featuring central non-enhancing areas indicative of necrosis or scarring. CECT, contrast-enhanced computed tomography

Ultrasonography revealed a large, mixed echogenic mass in the left kidney. Contrast-enhanced computed tomography (CECT) showed a 14 × 10 × 10 cm renal mass at the upper pole with mixed echogenicity and contrast enhancement (>15 HU) (Figure 1). The working diagnosis was renal cell carcinoma, and metastasis staging was negative.

**Figure 2:**
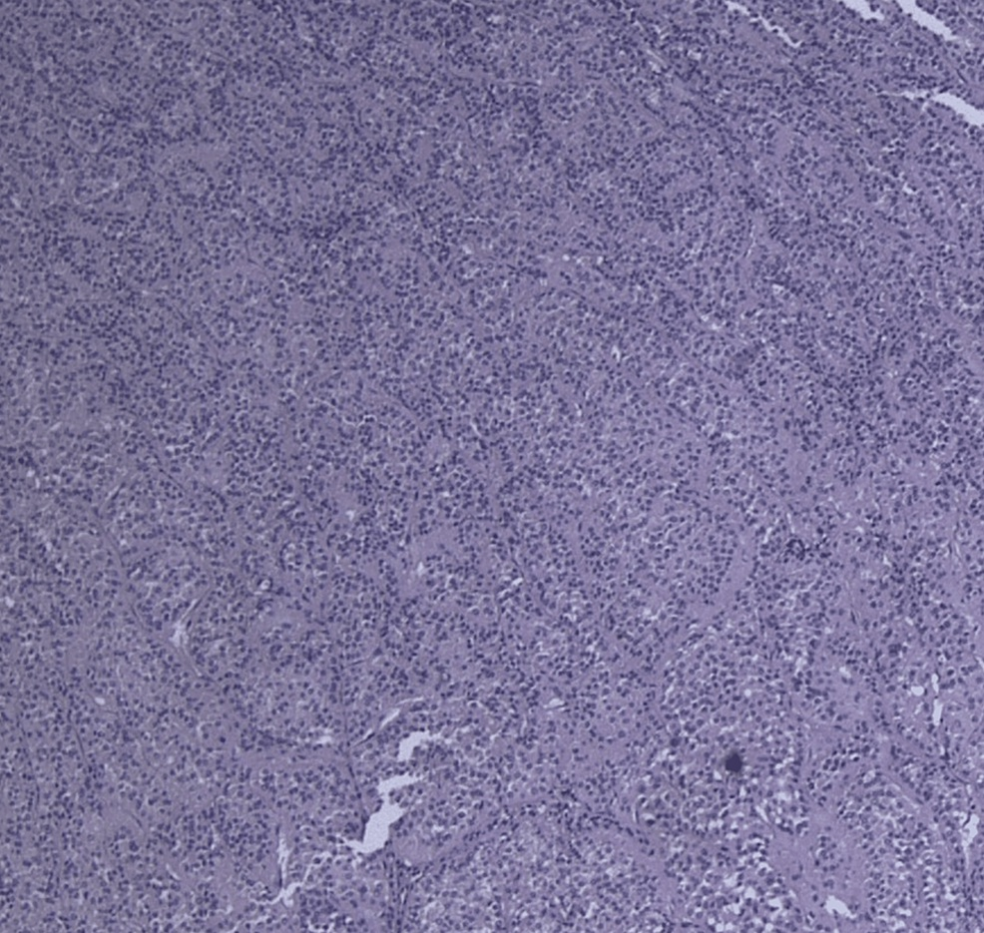
Microscopic analysis of the hematoxylin and eosin-stained tumor shows the classic architecture of an oncocytoma with large eosinophilic cells with regular nuclei arranged in nests.

The patient underwent a successful elective left radical nephrectomy through a subcostal Kocher incision without intraoperative complications. Postoperative recovery was unremarkable, and the patient was discharged on the fifth postoperative day. Histopathologic examination revealed a 13.5 × 10 × 9.8 cm encapsulated light brown tumor weighing 1,092 grams. Microscopically, the cells showed eosinophilic cytoplasm and regular nuclei. Immunohistochemical findings confirmed the diagnosis of renal oncocytoma (Figure 2).

## Discussion

Renal oncocytomas were first described by Zippel in 1942 and were initially considered malignant [1]. They are more common in men and peak in the sixth to seventh decade of life. Oncocytomas may be bilateral in 4-5% of cases and multifocal in up to 13% [2].

Oncocytomas arise from the neoplastic growth of intercalated epithelial cells of the collecting duct [2]. Specific genetic abnormalities have been associated with renal oncocytomas, including translocations in 11q13, loss of chromosome Y, loss of chromosome 1, and loss of heterozygosity on chromosome 14q. In addition, oncocytomas frequently occur in patients with Birt-Hogg-Dube syndrome [3].

Although most oncocytomas are incidental findings, they can occasionally be symptomatic. Hematuria, flank pain, and a palpable mass are the most common symptoms when present. Cross-sectional imaging (CT or MRI) may identify a renal tumor; the presence of a central scar and hypervascularity, although not pathognomonic, should raise suspicion of oncocytoma. Unfortunately, there are no definitive radiologic criteria to distinguish an oncocytoma from a malignant lesion, and oncocytomas may coexist with renal cell carcinoma in 10-32% of cases [2].

Fine-needle aspiration and tumor core biopsy are often nondiagnostic because the pathologic features of oncocytomas may resemble renal cell carcinoma, particularly chromophobe renal cell carcinoma. In extensive lesions, the possibility of sampling error should be considered when performing a biopsy [4]. Suspicious renal lesions should be surgically removed in most cases, as definitive diagnosis requires histopathologic analysis, including immunohistochemical panels.

Grossly, oncocytomas are brownish and well-circumscribed with a capsule. They consist of round to polygonal cells with eosinophilic cytoplasm and uniform nuclei with distinct nucleoli. Immunohistochemically, oncocytomas stain positive for CD117 and S100 calcium-binding protein A1 and negative for CK7, vimentin, and CD10 [5].

Renal oncocytomas are usually small, averaging 4 to 7 cm in size. Large oncocytomas larger than 10 cm, as in our case, are rare, and to our knowledge, only 14 cases have been described in the English-language literature (Table 1). The largest oncocytoma, measuring 27 × 20 × 15 cm, was described by Demos in 1988 [6]. 

**Table 1:** Cases of large oncocytomas in English literature.

No.	Reference	Date	Age	Sex	Location	Complaint	Size (cm)
1	Demos et al. [6]	1988	64	M	Right kidney	Abdominal mass	27×20×15
2	Banks et al. [8]	2001	57	M	Right kidney	Abdominal pain	21×18×15
3	Ponholzer et al. [9]	2002	63	F	Bilateral kidney	Weight loss	L:11×8.5×12 R:16×12×15
4	Kilic et al. [10]	2003	65	M	Left kidney	Abdominal pain	20×15×10
5	Sundararajan et al. [11]	2008	37	M	Right kidney	Abdominal mass and moderate hypertension	20
6	Shakarchi et al. [12]	2008	70	M	Right kidney	Asymptomatic	13×11×5.5
7	Akbulut et al. [13]	2009	25	F	Left kidney	Abdominal mass	25×16×12
8	Anastasiadis et al. [14]	2010	48	M	Left kidney	Abdominal discomfort	16.5×14
9	Ahmad et al. [15]	2011	61	M	Right kidney	Flank pain	25×16×16
10	Sussman et al. [16]	2017	-	M	Left kidney	Abdominal distention & shortness of breath	26
11	Dey et al. [17]	2019	59	F	Right kidney	Abdominal mass & pain	14×13×12
12	Badahori et al. [18]	2021	63	M	Right kidney	Flank pain	11×11×9
13	Qaid et al. [19]	2022	40	M	Left kidney	Intermittent abdominal pain	15×16×19,5
14	Çevik et al. [20]	2023	72	M	Right kidney	Asymptomatic	19×18×9.5
15	Current case	2023	84	W	Left kidney	Hematuria	14×10×10

Despite their size, oncocytomas have an excellent prognosis and rarely metastasize. Lesion growth should be considered, as recent studies have reported oncocytoma cells invading perinephric fat and/or venous branches. Oncocytomas should be included in the differential diagnosis of patients with renal mass regardless of their size. A high index of clinical suspicion and awareness is paramount to prompt and effective investigation, even in cases of patient-reported symptoms [7]. Because it is not possible to differentiate oncocytomas from renal cell carcinomas based on clinical, radiologic, or laboratory criteria, management may be challenging. Close surveillance might be an option for small lesions after biopsy confirmation. Still, a rapid increase in size should necessitate intervention for unidentified hybrid tumors or when the lesion becomes too large for ablative therapies or partial nephrectomy. In most cases, nephrectomy is the treatment of choice for renal oncocytomas. Partial nephrectomy is preferred for small oncocytomas, whereas radical nephrectomy, traditionally considered the standard treatment, is reserved for larger tumors such as ours.

## Conclusions

This case report highlights the diagnostic challenges and management of a giant renal oncocytoma in an elderly patient. Clinicians should consider oncocytomas regardless of size in the differential diagnosis of renal tumors. Surgical excision of suspicious renal lesions is often necessary because the definitive diagnosis requires histopathologic analysis and immunohistochemical studies. Despite their benign nature, careful surveillance and timely intervention are critical in managing oncocytomas, as they are prone to invasive growth and coexist with malignant lesions.
